# Zolbetuximab in the treatment of advanced gastric and gastroesophageal junction cancer: a systematic review

**DOI:** 10.3389/fimmu.2026.1870010

**Published:** 2026-07-16

**Authors:** Natalia Picheta, Julia Piekarz, Jakub Pobideł, Katarzyna Szklener, Magdalena Skórzewska

**Affiliations:** 1 Student Academic Group, Department of Clinical Oncology and Chemotherapy, Medical University, Lublin, Poland; 2 Department of Clinical Oncology and Chemotherapy, Medical University, Lublin, Poland

**Keywords:** anti – CLDN18.2, CLDN18.2, gastroesophageal cancer, zolbetuximab, zolbetuximab in GEJ

## Abstract

**Introduction:**

Advanced gastric and gastroesophageal junction (G/GEJ) adenocarcinomas are characterized by an aggressive course and a very poor prognosis. Due to the limited benefit of immunotherapy in patients with HER2-negative tumors, new therapeutic targets are sought. Zolbetuximab is a chimeric monoclonal antibody targeting the CLDN18.2 protein, which is overexpressed in 50–80% of gastric cancers.

**Materials and methods:**

This review is based on phase I–III clinical trials, including the pivotal SPOTLIGHT, GLOW, FAST, and ILUSTRO trials. The efficacy and safety of zolbetuximab in combination with chemotherapy were assessed as first-line treatment for adults with locally advanced or metastatic G/GEJ adenocarcinoma, HER2-negative, and highly CLDN18.2-expressing.

**Results:**

Early phase studies confirmed the clinical activity and tolerability of zolbetuximab. A Japanese phase I study demonstrated the safety and pharmacokinetic profile of zolbetuximab as monotherapy, establishing the recommended dose in subsequent studies. The phase II FAST study demonstrated that the addition of zolbetuximab to EOX chemotherapy significantly improved both PFS (HR 0.44; p=0.0005) and OS (HR 0.55; p=0.0001). The phase II ILUSTRO study demonstrated activity of zolbetuximab both as monotherapy (ORR 9%) and in combination with mFOLFOX6 (ORR 39%), supporting its advancement to phase III. These results were confirmed in two pivotal randomized phase III studies. In the SPOTLIGHT study, the addition of zolbetuximab to mFOLFOX6 reduced the risk of death by 25% (median OS 18.23 vs. 15.54 months), and in the GLOW study, the addition of zolbetuximab to CAPOX reduced the risk of death by almost 23% (median OS 14.39 vs. 12.16 months). The most common adverse events were nausea and vomiting, occurring primarily in the first treatment cycle.

**Conclusions:**

Zolbetuximab represents a significant advancement in the treatment of patients with advanced G/GEJ adenocarcinoma overexpressing CLDN18.2, addressing an important unmet clinical need. However, optimizing the stringent threshold for CLDN18.2 positivity (requiring staining in ≥75% of cells) and establishing a treatment regimen for patients with concurrent CLDN18.2 and PD-L1 expression remains a significant challenge. The review protocol was prospectively registered in the PROSPERO database (International Prospective Register of Systematic Reviews) under registration number CRD420261383764.

**Systematic review registration:**

https://www.crd.york.ac.uk/PROSPERO/, identifier CRD420261383764.

## 1 Introduction

Gastric and gastroesophageal junction (G/GEJ) adenocarcinomas remain significant oncological challenges, characterized by aggressive clinical courses and poor advanced-stage outcomes (1). They represent a major global health burden, ranking as the fifth most frequently diagnosed malignancy and the third leading cause of cancer-related mortality worldwide, accounting for over one million new cases and approximately 800,000 deaths annually. Although the overall incidence of distal gastric cancer has steadily declined across Europe and North America—largely attributed to *Helicobacter pylori* eradication and improved food preservation—this progress is offset by an epidemiological shift (2). Over the past several decades, Western populations have experienced a nearly 2.5-fold surge in the incidence of GEJ adenocarcinomas, a trend heavily driven by the escalating prevalence of gastroesophageal reflux disease (GERD) and the obesity epidemic (3). Compounding this shifting anatomical distribution is the non-specific early clinical presentation of these tumors. Because early–stage disease is largely asymptomatic, a disproportionate majority of patients—even in regions with established healthcare infrastructure—are diagnosed *de novo* with locally advanced, unresectable, or metastatic disease. The prognosis for this cohort remains poor, with historical population-based registries indicating a five-year survival rate of merely 2% for distant metastatic GEJ adenocarcinoma (4).

Although HER2-targeted agents and immune checkpoint inhibitors (ICIs) have markedly improved first-line treatment outcomes, their clinical benefit is restricted to specific molecular subsets. Patients with HER2-negative and programmed death ligand 1 (PD-L1) low tumors derive negligible benefit from modern immunotherapies, remaining reliant on conventional, suboptimally effective chemotherapy (5). This limited systemic efficacy highlights a critical unmet clinical need for novel, tumor-specific molecular targets for populations ineligible for established targeted therapies.

Physiologically, gastric epithelial architecture relies on tight junction proteins, particularly claudin-18 (CLDN18), to maintain cellular polarity and barrier integrity. Claudins are four-membrane proteins with two extracellular loops – ECL1 and ECL2 and intracellular N- and C-terminal regions in the cytoplasm (6). Transmembrane domains 1 and 4 are highly conserved, whereas domains 2 and 3 are more variable (7). While the CLDN18.1 isoform is expressed in pulmonary tissue, CLDN18.2 expression is strictly confined to differentiated gastric mucosa (8).

CLDN18.2 is structurally similar to other claudins. Its C-terminal cytoplasmic domain contains a PDZ-binding motif that facilitates interactions with tight junction adaptor proteins. These interactions are essential for maintaining tight junction integrity (9).

In healthy tissues, CLDN18.2 epitopes are buried within tight junction complexes, rendering them inaccessible to circulating macromolecules and the immune system. However, gastric carcinogenesis disrupts apical-basal polarity and cellular adhesion. This architectural breakdown and subsequent microenvironmental shifts promote epithelial-mesenchymal transition (EMT) and tumor invasiveness, ultimately leading to the extracellular unmasking of ectopic CLDN18.2 epitopes on the cancer cell surface (8). High CLDN18.2 expression correlates directly with the presence of CD8+ lymphocytes, neutrophils, and tumor-associated fibroblasts (CAFs). Although tumors with high CLDN18.2 harbor large numbers of CD8+ lymphocytes, patients with this expression often have poorer overall survival, which is explained by the lack of activity of these lymphocytes and their reduced expression of checkpoint receptors (10).

CLDN18.2 is aberrantly overexpressed in 50–80% of gastric cancers. This unique pathophysiological profile—robust surface expression on neoplastic cells coupled with spatial inaccessibility in normal tissues—provides a strong therapeutic rationale. Targeting CLDN18.2 enables the selective eradication of malignant clones while minimizing systemic off-target toxicity, presenting a distinct advantage over traditional oncological strategies (11).

Exploiting this paradigm, zolbetuximab emerged as a first-in-class, highly specific chimeric IgG1 monoclonal antibody targeting CLDN18.2. Rather than delivering a cytotoxic payload, zolbetuximab orchestrates a robust immune response within the tumor microenvironment. Upon binding to exposed epitopes, it triggers two critical effector pathways: antibody-dependent cellular cytotoxicity (ADCC) mediated by natural killer cells, and complement-dependent cytotoxicity (CDC), culminating in membrane attack complex (MAC) formation and tumor cell lysis (12).

## 2 Materials and methods

### 2.1 Study design

This systematic review was conducted to assess the efficacy and safety of zolbetuximab in the treatment of advanced G/GEJ adenocarcinoma. The analysis encompassed the drug’s comprehensive clinical development program, including various study types: early phase (phase I) studies, phase II studies (including multi-cohort and single-arm studies), and phase III randomized controlled trials (RCTs). Due to the specificity of the study population (required CLDN18.2 overexpression) and the relatively recent approval of the drug, the review is based on key published clinical trials evaluating this targeted therapy.

### 2.2 Eligibility criteria and PICO framework

The study focused on assessing the efficacy and safety of zolbetuximab therapy. The PICO model was used to structure and guide the literature review:

Population (P): The target population consisted of adult patients (aged ≥18 or ≥20 years, depending on the study) diagnosed with locally advanced (unresectable) or metastatic G/GEJ adenocarcinoma. A mandatory requirement for inclusion in the pivotal studies was HER2-negative status and confirmed CLDN18.2 protein expression.

Intervention (I): Interventions included the use of zolbetuximab (in various dosing regimens, including a loading dose of 800 mg/m², followed by a maintenance dose of 600 mg/m² or 400 mg/m²) as monotherapy, in combination with standard chemotherapy, or in combination with immunotherapy. Comparison (C): A comparative analysis was conducted comparing zolbetuximab plus chemotherapy with standard chemotherapy alone or chemotherapy plus placebo. Single-arm studies: In early-phase and single-arm studies, results were analyzed without comparison to a control group.

Outcome (O) - Efficacy: Evaluated primarily based on objective response rate (ORR), disease control rate (DCR), progression-free survival (PFS), and overall survival (OS). Safety was analyzed by assessing the incidence of treatment-related adverse events (TRAEs), including serious adverse events of grade ≥3 (particularly gastrointestinal and hematological events), events leading to treatment discontinuation, and treatment-emergent adverse events (TEAEs).

Studies published in English between 2019 and 2026 were included in the analysis. RCT and single-arm studies were included if they contained data on the use of zolbetuximab in the patient population in question. Exclusion criteria included review publications, reports of preclinical studies (both in animal and cellular models), abstracts without complete data (e.g., conference abstracts or posters that did not provide full results), and papers that did not include information on the efficacy or safety of zolbetuximab.

### 2.3 Search and selection process

The search terms in the databases: PubMed, Web of Science and Springer NatureLink registry covered the years 2019–2026 to provide the most up-to-date data. The systematic search was conducted using precise, database – specific search strings combining terms for the intervention and target disease. The exact search queries used were as follows:

- PubMed: (“Zolbetuximab” OR “Anti–CLDN18.2”) AND (“Gastroesophageal cancer”)
- Web of Science: (“Zolbetuximab” OR “Anti–CLDN18.2”) AND (“Zolbetuximab in GEJ” OR “Gastroesophageal cancer”)
- Springer NatureLink: (“CLDN18.2” OR “Anti–CLDN18.2”) AND (“Zolbetuximab in GEJ” OR “Gastroesophageal cancer”).

The final database search was conducted on April 12, 2026.

**Figure 1:**
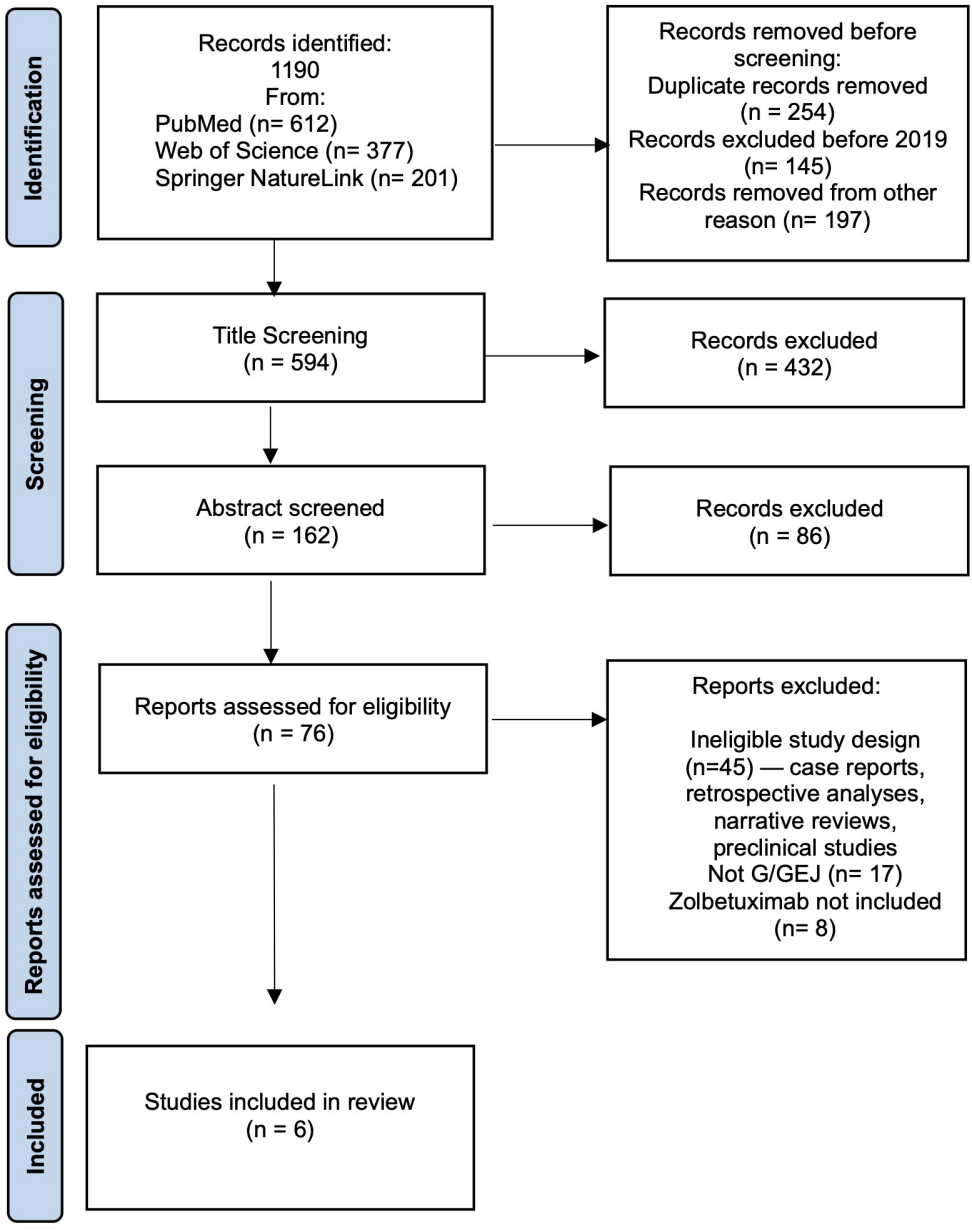
PRISMA (Preferred Reporting Items for Systematic Reviews and Meta-Analyses) flowchart for identifying, including, and excluding studies.

To ensure the completeness of the review, a manual search of the results and an evaluation of the bibliographies of the selected articles were also conducted. Two researchers (N.P. and K.K.) independently searched the databases and critically appraised the selected articles. Any discrepancies were resolved through verification by three other authors (J.P., J.Pob. and M.S.). A systematic database search identified 1,190 records. Prior to screening, 596 records were removed: 254 duplicates, 145 records published before 2019, and 197 records excluded by automated database filters – non – English language publications, non – journal article types. The remaining 594 records were subjected to title screening, of which 432 were excluded. Of the 162 abstracts searched, 86 full-text reports were excluded as their main topics were deemed irrelevant to specific research questions, leaving 76 reports for eligibility assessment. After excluding 70 reports (45 due to ineligible study design, 17 due to non-G/GEJ population, and 8 due to lack of zolbetuximab), 6 studies met all inclusion criteria and were included in the final analysis. The review protocol was prospectively registered in the PROSPERO database (International Prospective Register of Systematic Reviews) under registration number CRD420261383764. The comprehensive data selection and identification process is illustrated in **Figure 1**.

### 2.4 Data extraction

The review included one nonrandomized, open-label, multi-cohort phase I study, two phase II studies (ILUSTRO reported as two separate publications covering different cohorts), and two RCTs. Data extraction was performed independently by two reviewers using a standardized data extraction form. Extracted data included study characteristics (author, year, study design), patient demographics (total number of patients), intervention details (zolbetuximab, regimen), and outcome measures related to efficacy (ORR, PFS, OS, DoR, best overall response (BOR), stable disease (SD), progressive disease (PD), and safety (assessment of the incidence of TEAEs and TRAEs, including serious adverse events grade ≥3 and events leading to treatment discontinuation). Any discrepancies were resolved through discussion or consultation with other reviewers.

### 2.5 Risk of bias assessment of included studies

The included studies consisted of RCTs and early-phase multi-cohort studies (including single-arm studies).

For RCTs, the risk of bias was assessed using the updated Cochrane Risk of Bias tool for randomized controlled trials (RoB 2.0). This tool assesses five domains: (1) bias due to randomization, (2) bias due to deviations from intended interventions, (3) bias due to missing outcome data, (4) outcome measurement bias, and (5) bias in reporting outcome selection. Each domain was rated as “low risk,” “some concern,” or “high risk,” and an overall risk of bias score was assigned to each study. Both phase III trials (SPOTLIGHT and GLOW), as well as the phase II trial (FAST), demonstrated an overall low risk of bias across all evaluated domains. The results of this assessment are presented in **Table 1**.

**Table 1:** Risk of bias assessment for randomized controlled trials using the Cochrane RoB 2.0 tool.

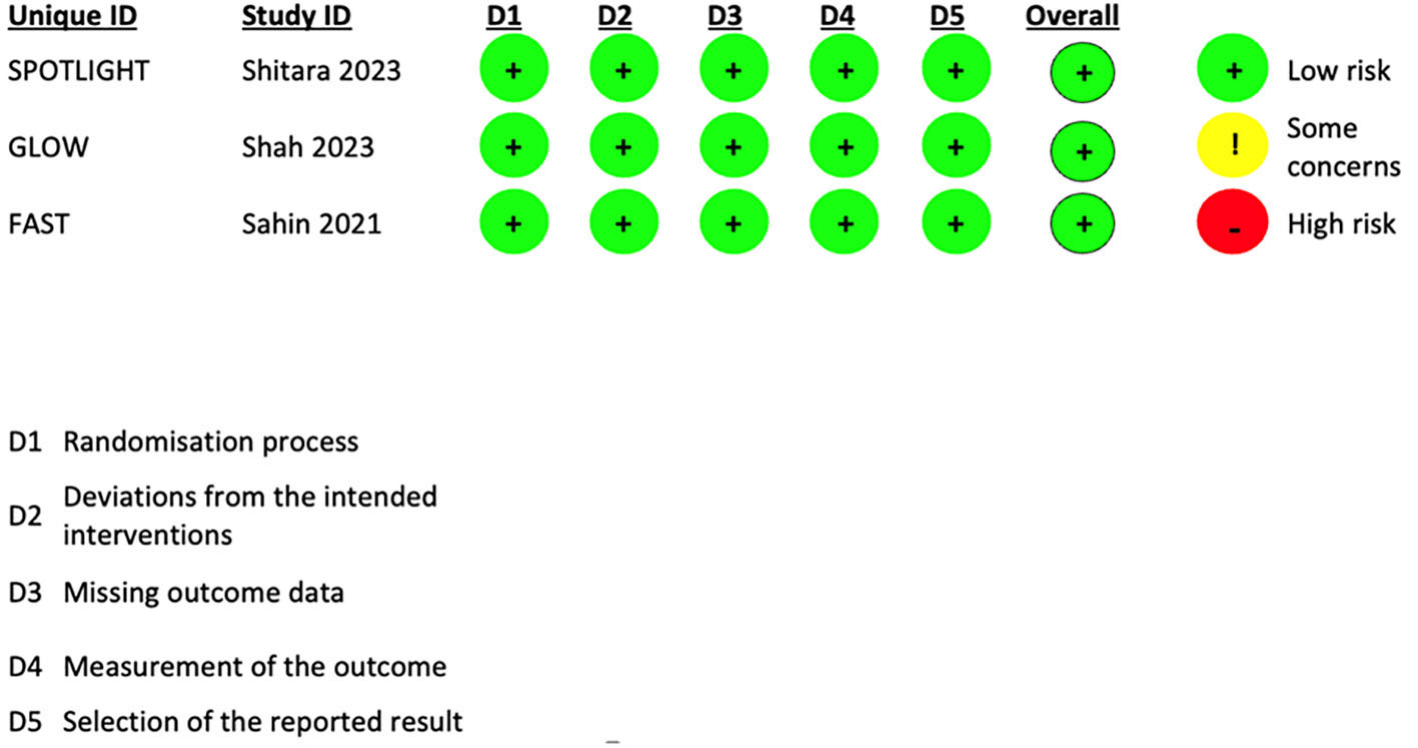

For single-arm phase I and II studies, methodological quality was assessed using the JBI Critical Appraisal Checklist for Quasi-Experimental Studies, which showed that all three non-randomized trials (Shitara et al., 2022; Klempner et al., 2023; Shitara et al., 2026) demonstrated high methodological quality, satisfying 7 out of 8 applicable criteria, as the control group criterion was not applicable to these single-arm designs. Studies meeting ≥70% of the relevant checklist criteria were considered high-quality. Two reviewers (N.P. and K.K.) independently assessed the risk of bias for all included studies. Discrepancies were resolved through discussion with three additional authors (J.P., J.Pob., and M.S.).

## 3 Zolbetuximab

Zolbetuximab is a chimeric immunoglobulin G1 (IgG1) monoclonal antibody that specifically targets CLDN18.2. It contains variable regions derived from the murine anti-human CLDN18.2 monoclonal antibody fused to human IgG1 constant regions (13). Its mechanism of action involves binding to CLDN18.2 on the surface of tumor cells, engaging immune effector mechanisms —primarily ADCC and CDC. In ADCC, the antibody binds to the CLDN18.2 protein, recruiting natural killer (NK) cells via Fc gamma receptors (FcγRs) located on the NK cell surface, which recognize the Fc region of the antibody. This triggers the release of cytotoxic granules — perforins and gransins —that lead to apoptosis in target cells (14). In CDC, binding of zolbetuximab to CLDN18.2 activates the complement pathway, allowing the C1 complex to bind to the Fc region of the antibody. This triggers a proteolytic cascade that leads to MAC formation, causing disruption and lysis of the tumor cell membrane. In addition to direct immune cytotoxicity, zolbetuximab exhibits synergistic effects with chemotherapy (14).

**Figure 2:**
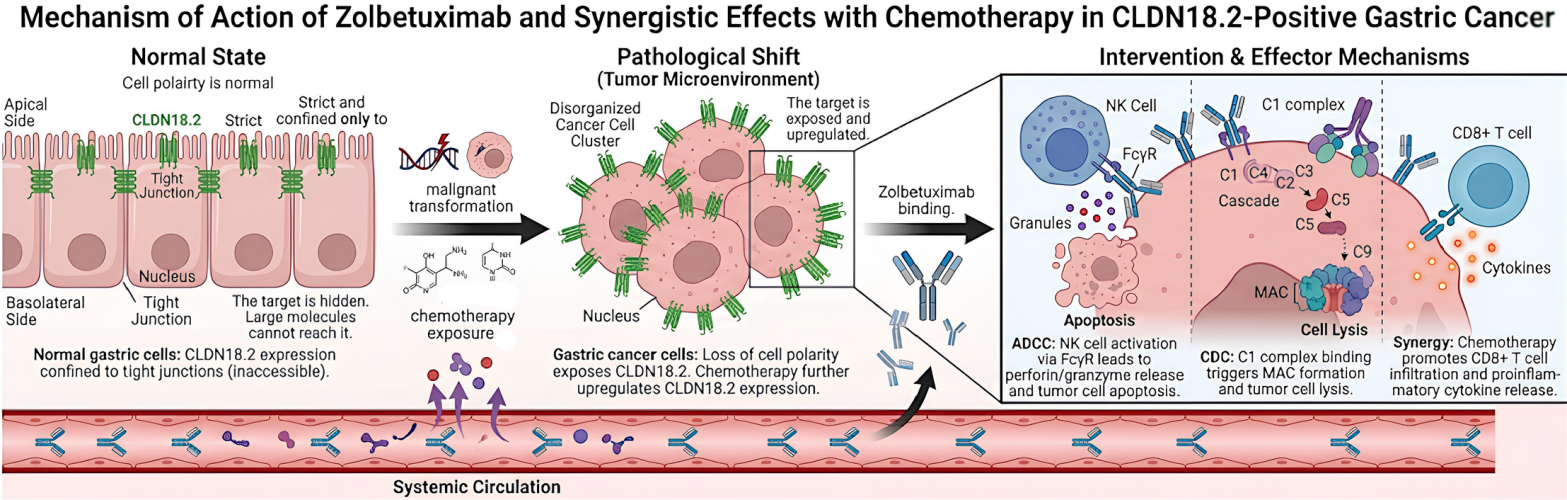
Mechanism of action of zolbetuximab (16).

When combined with fluoropyrimidine- and platinum-based chemotherapy, zolbetuximab can also promote T cell infiltration and stimulate the release of proinflammatory cytokines. Furthermore, chemotherapy can enhance zolbetuximab’s efficacy by increasing CLDN18.2 expression on tumor cells and promoting CD8+ T cell infiltration into the tumor microenvironment (15). **Figure 2** illustrates the mechanism of action of zolbetuximab.

Zolbetuximab binds to the CLDN18.2 protein, which is exposed on the surface of cancer cells, triggering antibody-dependent cytotoxicity (ADCC) and complement-dependent cytotoxicity (CDC) mechanisms, leading to tumor destruction. Additionally, the drug demonstrates synergistic activity with chemotherapy, which increases expression of the target protein and stimulates an anti-tumor immune response (16).

On October 18, 2024, the Food and Drug Administration (FDA) approved zolbetuximab based on the phase III SPOTLIGHT and GLOW studies for use with fluoropyrimidine and platinum in first-line treatment of adults with locally advanced, unresectable or metastatic G/GEJ adenocarcinoma. At the same time, the FDA also approved the VENTANA CLDN18 (43-14A) RxDx test (Ventana Medical Systems, Inc./Roche Diagnostics) as a diagnostic device to aid in identifying patients with gastric or gastrointestinal adenocarcinoma who may be eligible for zolbetuximab treatment (17). The European Medicines Agency (EMA) approved zolbetuximab for marketing ahead of the FDA on September 19, 2024. The drug is now under additional monitoring (18). The drug continues to maintain orphan drug status.

## 4 Research

### 4.1 Phase I study

The study enrolled 18 adult Japanese patients aged ≥20 years with histologically confirmed locally advanced or metastatic G or GEJ cancer (19).

Patients were divided into two phases: a safety arm: arm A (n=3) and arm B (n=3), and an expansion arm (n=12). Eligibility for the study procedure was contingent upon exhaustion of available standard therapeutic options or the lack of clinical predisposition to implement them, based on the investigator’s reliable assessment. Furthermore, precise biomarker criteria were introduced, verified by central immunohistochemical testing: patients demonstrating any intensity of CLDN18.2 membrane protein staining were included in the safety arm, while only patients with high CLDN18.2 expression, characterized by a staining intensity of ≥2+ in at least 75% of tumor cells, were included in the expansion arm (19). Patients in the safety arm A and the expansion arm received intravenous zolbetuximab at a loading dose of 800 mg/m² body surface area on the first day of the first cycle, followed by a maintenance dose of 600 mg/m² administered at three-weekly intervals (Q3W). In the safety arm B, investigators implemented monotherapy with a fixed dose of 1000 mg/m² of the study antibody administered in a similar three-weekly schedule (19).

In the subgroup of 17 patients with measurable tumor lesions, the best overall response (BOR) was SD in 64.7% of patients and progression (PD) in the remaining 35.3% of patients. This translated into a cumulative disease control rate (DCR) of 64.7%. Survival analysis of the combined arm A and expansion cohorts revealed a median PFS of 2.6 months with a median OS of 4.4 months. In the separate arm B cohort, a median PFS of 1.7 months and a median OS of 6.4 months were observed (19). No dose-limiting toxicities or treatment-related adverse events (TRAEs) of grade ≥3 were reported. 94.4% of subjects experienced grade 1–2 adverse events related to zolbetuximab, the most common of which were nausea (61.1%), epigastric pain (44.4%), and vomiting (38.9%).

### 4.2 Phase II studies

#### 4.2.1 FAST

A total of 252 patients were enrolled and randomized in the study. Allocation to the individual arms was as follows: arm 1 (control): 84 patients; arm 2 (experimental): 77 patients; arm 3 (exploratory, added to the protocol at a later stage): 85 patients. The primary analysis was based on a population of 161 patients from the first two arms. Only adult patients aged ≥18 years with histologically confirmed, locally advanced and inoperable, recurrent or metastatic G/GEJ or esophageal adenocarcinoma (EC) were eligible for the study. Meeting a precise biomarker criterion was also mandatory for participation, requiring moderate or strong CLDN18.2 protein expression – defined as a staining intensity of (2+) or (3+) – in at least 40% of the analyzed tumor cells (20). The baseline regimen was EOX chemotherapy, including epirubicin at a dose of 50 mg/m², oxaliplatin at a dose of 130 mg/m², and capecitabine at a dose of 625 mg/m² twice daily. Patients assigned to arm 1 received only the EOX Q3W protocol. In arm 2, combination therapy was implemented, supplementing standard chemotherapy with intravenous infusions of zolbetuximab at a loading dose of 800 mg/m² on the first day of the first cycle, followed by a maintenance dose of 600 mg/m² in the Q3W regimen. For arm 3, a protocol was defined based on the combination of the EOX regimen with zolbetuximab at a higher, fixed dose of 1000 mg/m², administered similarly every three weeks (16). Clinical efficacy analysis showed that in the overall population (comparing Arm 2 with Arm 1), the addition of zolbetuximab to EOX chemotherapy resulted in a highly statistically significant extension of median PFS from 5.3 to 7.5 months, which from a statistical perspective means a reduction in the risk of disease progression or death by as much as 56% (HR = 0.44; 95% CI: 0.29–0.67; p < 0.0005). A similar benefit was observed in overall survival (OS), where the studied intervention extended the median PFS from 8.3 to 13 months, reducing the risk of death by 45% (HR = 0.55; 95% CI: 0.39–0.77; p < 0.0005). In a distinct subpopulation of patients with moderate to strong biomarker expression (≥70% of tumor cells expressing CLDN18.2), clinical benefit was further amplified. In this cohort, the parameters achieved HR = 0.38 for PFS and HR = 0.50 for OS (20).

Most adverse events (AEs) were mild or moderate, with grades 1-2. There was no significant overall increase in the incidence of serious adverse events (grade >3) compared to the chemotherapy-alone group. Gastrointestinal symptoms dominated the toxicity profile: nausea (81.8% in patients in Arm 2 vs. 76.2% in Arm 1) and vomiting (67.5% vs. 54.8%). The incidence of serious adverse events was lower in patients receiving zolbetuximab in combination with EOX (24.7%) compared to patients receiving EOX alone (32.1%) (20).

#### 4.2.2 ILUSTRO

The study included 54 patients aged ≥18 years with histologically confirmed advanced G/GEJ adenocarcinoma. CLDN18.2-positive tumors were determined by central immunohistochemistry. A positive result was defined at two levels: high expression: ≥75% of tumor cells exhibiting moderate to strong immunohistochemical (IHC) staining of the cell membrane (2+/3+) and intermediate expression: ≥50% to <75% of tumor cells with moderate to strong staining, measurable disease according to the Response Evaluation Criteria in Solid Tumors (RECIST 1.1), and normal organ function (21). Cohorts and specific inclusion criteria by cohort are presented in **Table 2**.

**Table 2:** Detailed description of the cohorts from the ILUSTRO study (21).

Cohort	Number of patients	Treatment used	Inclusion criteria
1A	30	Zolbetuximab monotherapy	≥3 prior lines of therapy required, high or intermediate CLDN18.2 expression accepted
2	21	Zolbetuximab + mFOLFOX6	Did not require prior systemic therapy for advanced/metastatic disease (first-line treatment); HER2-negative tumors
3A	3	Zolbetuximab + pembrolizumab	Third/later line treatment; high/intermediate CLDN18.2 expression was accepted in cohort 3A

Cohort 3B (requiring PD-L1 combined positive score (CPS) ≥1) and cohort 4 (zolbetuximab + mFOLFOX6 + nivolumab) were planned but not included in this publication.

Zolbetuximab was administered in all cohorts using an identical regimen: an intravenous loading dose of 800 mg/m² in cycle 1, followed by a maintenance dose of 600 mg/m² Q3W. FOLFOX6 was administered at doses of oxaliplatin 85 mg/m², leucovorin 400 mg/m², and 5-fluorouracil at a bolus of 400 mg/m² and a continuous infusion of 2400 mg/m² every 2 weeks. Pembrolizumab was administered at a fixed dose of 200 mg every 3 weeks (21).

Cohort 2 demonstrated the highest efficacy. The objective response rate (ORR) was 71.4% (95% CI: 47.82–88.72), and the DCR reached 100% (95% CI: 83.89–100). Median progression-free survival (PFS) was 17.8 months (95% CI: 8.05–25.69). In cohort 1, the ORR was 0% and the DCR was 44.4% (95% CI: 25.48–64.67). Median PFS was only 1.54 months (95% CI: 1.31–2.56), while median OS was 5.62 months (95% CI: 2.27–11.53).

In cohort 3A, the ORR was 0% and the DCR was 66.7% (95% CI: 9.43–99.16). The median PFS was 2.96 months (95% CI: 1.48–4.44).

TEAEs were as follows: in Cohort 1A, the most common adverse events of any grade were nausea (63.3%), abdominal pain (40%), and vomiting (36.7%). Grade ≥3 events occurred in 50% of patients; the most common were anemia, abdominal pain, and hypertension (10% each).

Cohort 2: Nausea was reported by 90.5% of patients, and vomiting by 66.7%. Grade ≥3 events occurred in 95.2% of patients, with hematologic toxicities predominating: neutropenia (23.8%) and decreased neutrophil count (28.6%). Serious nausea and vomiting (grade ≥3) occurred in 4.8% and 9.5% of patients, respectively. Cohort 3A: The most common adverse events reported were nausea, constipation, fever, and decreased appetite (66.7% each). A grade ≥3 adverse event, a decrease in neutrophil count, was reported in 1 patient (33.3%). No immune-related toxicity was observed (21).

#### 4.2.3 Continuation of ILUSTRO – cohort 4

This study describes cohort 4 of the ILUSTRO study. Seventy-seven patients were enrolled. The general inclusion criteria were the same as for the previous study, while cohort 4-specific criteria included no prior systemic treatment for metastatic disease. Patients were divided into two cohorts: 4A (n=6), an initial safety assessment phase, and 4B (n=71), an expansion phase. Zolbetuximab was administered at a loading dose of 800 mg/m² in cycle 1, followed by a maintenance dose of 400 mg/m² in subsequent cycles, nivolumab at a fixed dose of 240 mg administered as a 30-minute intravenous infusion, and mFOLFOX at the same dose as in the previously described study (22).The median PFS in the entire 4B cohort was 14.8 months (95% CI: 8.3 – not reached [NE]). In the identified subpopulation of patients with high CLDN18.2 expression, this parameter was higher, reaching 18.0 months (95% CI: 11.1 – NE). The best results were observed in patients with concurrent high CLDN18.2 expression and a PD-L1 CPS ≥1, in whom the median PFS was as high as 23.6 months.

Median OS at a follow-up time of 15.4 months was 18.0 months (95% CI: 13.6 – NE) for the entire 4B cohort. For the key subpopulation with high CLDN18.2 expression, median survival has not yet been reached (NE; 95% CI: 13.7 – NE) due to data immaturity. In Cohort 4B patients with measurable tumor lesions, the ORR was 62.1% (95% CI: 48.4–74.5). In the group selected for high CLDN18.2 expression, this rate was 68.1% (95% CI: 52.9–80.9), with a DCR of 89.7%. Among patients with dually high CLDN18.2 expression (high + CPS ≥1), the ORR reached a noTable 74.2% (22).

Severe TEAEs (grade ≥3) occurred in 66.2% of Cohort 4 participants, with decreased neutrophil counts predominating (32.5%). Serious adverse events led to permanent discontinuation of any protocol medication in 49.4% of patients. The most common adverse events of any grade were nausea (80.5%), decreased appetite (72.7%), peripheral sensory neuropathy (45.5%), decreased neutrophil count (45.5%) and vomiting (37.7%) (22).

### 4.3 Phase III studies

#### 4.3.1 SPOTLIGHT

This randomized, double-blind, phase III study enrolled 565 patients. Inclusion criteria included age ≥18 years, locally advanced (unresectable) or metastatic G/GEJ adenocarcinoma, no prior systemic therapy for advanced disease, HER2 negative status, Eastern Cooperative Oncology Group (ECOG) performance status of 0 or 1, and positive CLDN18.2 status, defined very stringently as the presence of moderate or strong membranous staining in ≥75% of analyzed tumor cells by immunohistochemistry (23).

Patients were divided into two groups: an experimental arm (n = 283), of which 279 received at least one dose of treatment, and a control arm (n = 282), of which 278 received at least one dose of treatment. In the experimental arm, patients received intravenous zolbetuximab at a loading dose of 800 mg/m², followed by a maintenance dose of 600 mg/m² every 3 weeks. This therapy was combined with the mFOLFOX6 regimen—folinic acid 400 mg/m², fluorouracil 400 mg/m² bolus and 2400 mg/m² by continuous infusion, and oxaliplatin 85 mg/m²—administered every 2 weeks. In the control arm, placebo was administered every 3 weeks in combination with the standard mFOLFOX6 regimen administered every 2 weeks. The median follow-up was 12.94 months in the zolbetuximab group compared to 12.65 months in the placebo group (23).The primary endpoint was PFS. PFS in the experimental arm was 10.61 months, and 8.67 months in the control arm. This translates to a 25% reduction in the risk of disease progression or death (HR = 0.75; 95% CI: 0.60–0.94; p = 0.0066). OS in the experimental arm was 18.23 months (95% CI: 16.43–22.90), and in the control arm was 15.54 months (95% CI: 13.47–16.53). The risk of death was reduced by 25% (HR = 0.75; 95% CI: 0.60–0.94; p = 0.0053). ORR was similar in both groups: 61% in the zolbetuximab group versus 62% in the control group. The most common adverse events, which occurred significantly more frequently in the experimental TAEA group, were gastrointestinal: nausea (82% for zolbetuximab vs. 61% for placebo), vomiting (67% vs. 36%), and decreased appetite (47% vs. 33%). Grade ≥3 TAEAs occurred in 87% (242/279) of patients in the zolbetuximab group and in 78% (216/278) of patients in the placebo group (23).

The most common TRAEs were nausea: 72.4% in the zolbetuximab group vs. 54% in the placebo group, vomiting: 64.9% vs. 30.2%, and decreased appetite: 40.5% vs. 27.7%.

TRAEs led to discontinuation of zolbetuximab in 14% of patients compared to 2% in the placebo group. Deaths directly related to treatment (grade 5 toxicity) were rare and occurred with similar frequency in both arms: 5 patients (2%) in the experimental group and 4 patients (1%) in the control group (23).

#### 4.3.2 GLOW

The GLOW trial was a phase III, double-blind RCT that enrolled 507 patients. Inclusion criteria were: adult patients with locally advanced or metastatic G/GEJ adenocarcinoma, no prior systemic treatment for advanced disease, negative HER2 receptor status, ECOG 0–1, and high CLDN18.2 expression defined as moderate or strong membranous staining in ≥75% of tumor cells by immunohistochemistry (24).Patients were divided into two groups: the experimental group (n = 254) received an intravenous loading dose of 800 mg/m² on day 1 of cycle 1, followed by 600 mg/m² in subsequent cycles with CAPOX chemotherapy: oral capecitabine (1000 mg/m² twice daily on days 1-14) and intravenous oxaliplatin (130 mg/m² on day 1 of each cycle), and the control group (n = 253) – placebo with CAPOX (24).

The primary endpoint was PFS. Median PFS was statistically significantly prolonged from 6.80 months in the placebo group to 8.21 months in the zolbetuximab group. This translates into a reduction in the risk of progression or death by approximately 31% (HR = 0.687; 95% CI: 0.544–0.866; p = 0.0007). Median OS increased from 12.16 months (control group) to 14.39 months (experimental group), which represents a reduction in the risk of death by almost 23% (HR = 0.771; 95% CI: 0.615–0.965; p = 0.0118). ORRs in the overall population were very similar: 42.5% for zolbetuximab and 40.3% for placebo. However, in the subpopulation of patients with measurable tumor lesions, the ORR was 53.8% for zolbetuximab versus 48.8% for placebo (24). Grade ≥3 TEAEs occurred in 72.8% of patients in the experimental arm and in 69.9% of patients in the control group, with an overall incidence of 98.8% and 98%, respectively. The only adverse events of any grade that showed a >10% difference between groups were nausea (68.5% zolbetuximab vs. 50.2% placebo) and vomiting (66.1% vs. 30.9%), which typically occurred in the first cycle and resolved in subsequent cycles.

The most common TRAEs were nausea (65.7% in the zolbetuximab group vs. 46.6% in the placebo group), vomiting (64.2% vs. 25.7%), decreased appetite (35.4% vs. 26.1%), and diarrhea (29.5% vs. 30.1%). TRAEs led to permanent discontinuation of zolbetuximab in 7.1% of patients (compared to 4.4% in the placebo group). Fatal toxicity related to study treatment was rare: 6 patients (2.4%) in the zolbetuximab arm and 7 patients (2.8%) in the placebo group (24). **Table 3** summarizes all studies described above.

**Table 3:** Summary of studies included in the review (19–24).

Author and year	Type of research	Population	Intervention	Control group	Results
Shitara K. et al. 2022	Nonrandomized, open-label, multi-cohort Phase I study	17	Safety Part: Arm A: Zolbetuximab 800 mg/m² loading dose → 600 mg/m² Arm B: Zolbetuximab 1000 mg/m² Expansion Part: Same dosing as Arm A: 800/600 mg/m²	–	Arm A + Expansion combined (n = 15): mPFS 2.6 months, mOS 4.4 months Arm B alone (n = 3): mPFS 1.7 months, mOS 6.4 months
Sahin, U. et al. 2021	RCT phase II	252	EOX + zolbetuximab 800 mg/m² on the first day of the first cycle, maintenance dose 600 mg/m² in the Q3W regimen	EOX: epirubicin 50 mg/m², oxaliplatin 130 mg/m², and capecitabine 625 mg/m² twice daily.	PFS: Zolbetuximab + EOX: median 7.5 months. EOX: median 5.3 months 56% reduction in the risk of disease progression or death (HR = 0.44; 95% CI: 0.29–0.67; p < 0.0005). OS: zolbetuximab + EOX: median 13.0 months. EOX: median 8.3 months. 45% reduction in the risk of death (HR = 0.55; 95% CI: 0.39–0.77; p < 0.0005).
Klempner SJ, et al. 2023	Phase II, open-label, multicohort trial	51 (cohort 1A,2,3A)	Cohort 1A: Zolbetuximab monotherapy (800 mg/m² loading dose, then 600 mg/m² every 3 weeks) Cohort 2: Zolbetuximab (800/600 mg/m²) + mFOLFOX6 (oxaliplatin 85 mg/m², leucovorin 400 mg/m² and 5-fluorouracil bolus 400 mg/m² and continuous infusion 2400 mg/m² every 2 weeks Cohort 3A: Zolbetuximab (800/600 mg/m²) + pembrolizumab (200 mg every 3 weeks)	–	Cohort 1A: ORR = 0%, DCR = 44.4%, median PFS = 1.54 months, median OS = 5.62 months. Cohort 2 (highest efficacy, 1st line): ORR = 71.4%, DCR = 100%, median PFS = 17.8 months. Cohort 3A: ORR = 0%, DCR = 66.7%, median PFS = 2.96 months.
Shitara et al., 2026	Phase II, open-label, multicohort trial	77	Zolbetuximab: 800 mg/m² in the first cycle, and in the subsequent cycles 400 mg/m² + nivolumab 240 mg + mFOLFOX (doses the same as in Klempner’s study)	–	Overall Cohort 4B population: ORR = 62.1%, DCR = 89.7%, median PFS = 14.8 months, median OS = 18.0 months. In the subpopulation with high CLDN18.2 expression (≥75%): ORR = 68.1%, median PFS = 18.0 months, median OS = not reached. In the subpopulation with dual expression (CLDN18.2 ≥75% and PD-L1 CPS ≥1): ORR = 74.2%, median PFS = 23.6 months.
Shitara K. et al., 2023	RCT double blind, phase III	565	Zolbetuximab intravenously: 800 mg/m², followed by a maintenance dose of 600 mg/m² every 3 weeks + mFOLFOX6 – folinic acid 400 mg/m², fluorouracil 400 mg/m² in bolus and 2400 mg/m² in continuous infusion, oxaliplatin 85 mg/m²) every 2 weeks.	Placebo + mFOLFOX at the same dose as in the study arm	Zolbetuximab + mFOLFOX: PFS: 10.61 months (HR = 0.75; p = 0.0066) mOS: 18.23 months (HR = 0.75; p = 0.0053) ORR = 61% Placebo + mFOLFOX: PFS: 8.67 months mOS: 15.54 months (95% Cl: 13,47–16,53). ORR = 62%
Shah MA. et al., 2023	RCT, double blind, phase III	507	Zolbetuximab: 800 mg/m², in subsequent cycles at a dose of 600 mg/m² with CAPOX chemotherapy: capecitabine (1000 mg/m², oxaliplatin (130 mg/m²)	Placebo + CAPOX	Zolbetuximab: Median PFS: 8.21 months. Approximately 31% reduction in the risk of progression or death (HR = 0.687; 95% CI: 0.544–0.866; p = 0.0007) Median OS: 14.39 months 23% reduction in the risk of death (HR = 0.771; 95% CI: 0.615–0.965; p = 0.0118) ORR overall: 42,5% Placebo: Median PFS 6.80 months Median OS 12.16 months Overall ORR: 40.3%

## 5 Discussion

The treatment of G/GEJ adenocarcinoma remains a significant and important clinical issue. Given the growing epidemiological burden and the limitations of conventional cytotoxic therapy in late-stage disease, systemic integration of novel targeted agents is clinically warranted. In this context, zolbetuximab provides a highly specific therapeutic approach for patients with advanced disease.

In the current therapeutic landscape for advanced, HER2-negative G/GEJ adenocarcinomas, frontline treatment algorithms are heavily stratified by biomarker expression, specifically PD-L1 levels. According to the European Society for Medical Oncology (ESMO) Clinical Practice Guidelines, conventional platinum- and fluoropyrimidine-based doublet chemotherapy remains the foundational cytotoxic backbone (25). However, recent practice-changing data from large-scale phase III randomized controlled trials have redefined the standard of care through the integration of ICIs. Specifically, the global CheckMate-649 trial demonstrated a profound, statistically significant survival benefit when incorporating the anti-PD-1 antibody nivolumab into standard first-line chemotherapy, establishing this combination as the primary recommendation for tumors exhibiting a high PD-L1 CPS, defined as CPS > 5 (26). This immunotherapeutic approach was further substantiated by the KEYNOTE-859 trial, which confirmed that adding pembrolizumab to standard first-line chemotherapy significantly prolongs survival outcomes in patients with a PD-L1 CPS > 1. Contemporary treatment algorithms mandate initial PD-L1 testing to guide the addition of ICIs, leaving conventional chemotherapy alone reserved primarily for populations with low or negative PD-L1 expression (27).

Zolbetuximab is a drug that targets a linearly restricted tight junction protein, not a canonical oncogenic factor (28). Unlike HER2 and EGFR, CLDN18.2 has no known role in oncogenic signaling; it functions as a “therapeutic anchor” that becomes available following loss of cell polarity during malignant transformation. This supports the concept of targeting non-oncogenic, tissue-specific proteins in solid tumors.

The included studies exhibit considerable clinical and methodological heterogeneity, spanning different study designs, treatment lines, chemotherapy backbones, and CLDN18.2 detection thresholds. To accurately contextualize these findings, it is essential to stratify the evidence. Early-phase exploratory studies (the Japanese Phase I, FAST, and ILUSTRO trials) were inherently heterogeneous but crucial for establishing the drug’s safety profile, identifying preliminary efficacy signals, and guiding the selection of the 800/600 mg/m² dosing regimen. These trials also explored various biomarker thresholds (e.g., ≥40% in FAST vs. ≥75% in ILUSTRO) and treatment settings. In contrast, the randomized evidence is derived from the Phase III trials (SPOTLIGHT and GLOW). These controlled studies focused strictly on the first-line setting with a unified, stringent biomarker threshold (≥75% positivity), providing more robust data regarding the efficacy of zolbetuximab in this specific population.

The primary efficacy analysis of the FAST trial was initially planned to include only arms 1 and 2 (n = 161), as arm 3 was added through a later protocol amendment. Importantly, arm 3 (zolbetuximab 1000 mg/m²) did not demonstrate a statistically significant OS benefit, justifying the selection of the 800/600 mg/m² dosing regimen in subsequent phase III studies (SPOTLIGHT, GLOW). Furthermore, the threshold for CLDN18.2 expression was ≥40% of tumor cells, significantly lower than the threshold used in the other studies. In the ILUSTRO study, the CLDN18.2 positivity threshold used in the study was ≥75% for high and ≥50% to <75% for intermediate, which is consistent with the definition later adopted in the pivotal phase III SPOTLIGHT study, which utilized the VENTANA CLDN18 (43-14A) RxDx assay with a positivity threshold of ≥75%. A large number of patients were screened—according to the SPOTLIGHT/GLOW screening data, approximately 38.4% of patients with G/GEJ adenocarcinoma tested ≥75% CLDN18.2 positivity, emphasizing the extensive screening efforts required to identify eligible patients. Results from the individual cohorts of the ILUSTRO study suggest that zolbetuximab works best in combination with chemotherapy in earlier lines of treatment (first line), rather than as monotherapy or in patients who had undergone intensive pretreatment. The significant difference between cohort 2 and the other cohorts helped support the phase III trials that ultimately led to regulatory approval. In the pembrolizumab cohort, an ORR of 0% and a median PFS of 3 months indicates limited efficacy, but only three patients were included in this cohort, making it difficult to draw firm conclusions. The lack of efficacy may be due to the intensive pretreatment rather than the combination strategy itself. Regarding adverse events in cohort 2, they may have been more pronounced due to the addition of chemotherapy. Importantly, in the SPOTLIGHT study, the ORR was similar in both groups, suggesting that zolbetuximab does not increase the likelihood of tumor response but rather prolongs the duration of response (9.0 vs. 6.8 months), contributing to the observed PFS and OS benefits.

After extended follow-up, final data presented at the American Society of Clinical Oncology (ASCO) Congress confirm the interim results: median PFS increased to 11.04 months in the zolbetuximab arm versus 8.94 months in the placebo group (HR = 0.734; p = 0.0024). Favorable outcomes were also observed in the Per-Protocol Set (PPS) population, with the median OS in the experimental group reaching 21.49 months compared to 16.39 months in the control group (HR = 0.687). This suggests that optimal treatment adherence and tolerability may further maximize the clinical benefit (29).

Regarding adverse events, the higher rates of nausea and vomiting with zolbetuximab are considered to be related to CLDN18.2 expression in the normal gastric mucosa. It is important to note that these were primarily grade 1–2, manageable adverse events.

Furthermore, an analysis of the SPOTLIGHT trial revealed that patients without prior gastrectomy had a higher rate of vomiting compared to patients with gastrectomy. This directly supports the role of CLDN18.2 expression in the stomach in mediating toxicity. Nausea and vomiting occur most frequently during the first and second infusions and then subside. This pattern is consistent with an initial immune-mediated injury to CLDN18.2-expressing gastric cells, with subsequent attenuation likely reflecting adaptation or exhaustion of CLDN18.2-expressing cells in the gastric mucosa.

A key unresolved question is the optimal threshold for CLDN18.2 positivity. For the SPOTLIGHT/GLOW assay, ≥75% of tumor cells with moderate or strong staining were required, which only covers ~36–38% of patients tested, however ADCs (e.g., CMG901) show activity at lower thresholds (≥20% of cells), in part because the cytotoxic payload kills adjacent CLDN18.2-negative cells (30).

For CAR-T therapy, a threshold of ≥40% is used, which expands eligibility to ~58% of patients (31). This raises the question: are we excluding patients who could benefit from CLDN18.2-targeted therapy (32)?

Paradoxically, the VENTANA CLDN18 assay detects both CLDN18 isoforms. However, this is not disqualifying in practice, as CLDN18.2 is expressed in gastric cancer, so it is understood that the common claudin is isoform 2 (33). Test performance analysis showed that positive agreement (PPA) and negative agreement (NPA) were both 100% when reproducible tests were performed using different antibody lots. In an interlaboratory reproducibility study conducted in three different laboratories by six pathologists, the average positive agreement (APA) was 91.5%, and the average negative agreement (ANA) was 90.7% (34). While the results are quite impressive, it is still an IHC test that is subject to visual assessment by the human eye, and the authors themselves emphasize that they were experienced pathologists.

Interestingly, high CLDN18.2 expression correlates with a “cooler” tumor microenvironment—fewer NK cells, M1 macrophages, and CD4+ T cells. However, paradoxically, the T-cell inflammation index (TIS) score is higher in tumors with high CLDN18.2 levels. This provides the basis for combining zolbetuximab with ICIs (as in the ILUSTRO Cohort 4 study and the ongoing LUCERNA study), which could potentially transform cold tumors into hot tumors via ADCC-dependent immune activation (35).

Exploratory biomarker data indicate that zolbetuximab treatment is associated with a rapid and significant reduction in ctDNA levels—even in patients who only achieved stable disease. This suggests that ctDNA may serve as an early marker of response, potentially guiding treatment decisions before radiographic changes become apparent (35).

Zolbetuximab is eliminated via catabolic degradation, bypassing the renal and hepatic pathways. A characteristic of monoclonal antibodies is their uptake by cells of the reticuloendothelial system (RES), such as macrophages, where it undergoes internalization and intracellular degradation in lysosomes to basic peptides and amino acids. The advantage of this mechanism is that drug clearance is independent of the function of these organs, ensuring a stable pharmacokinetic profile even in patients with renal or hepatic impairment. Furthermore, the slow rate of catabolic degradation results in a long half-life of zolbetuximab, allowing for less frequent dosing (36).

Determining HER2 status is crucial for selecting the appropriate treatment for gastric cancer. For HER2 – positive patients, treatment typically involves trastuzumab, chemotherapy, and pembrolizumab is administered. If it is negative, patients are tested for CLDN18.2 and PD-L1 to determine whether zolbetuximab or ICI should be added to chemotherapy. Recent data indicate that CLDN18.2 expression operates independently of these established biomarkers. This independence creates a significant clinical challenge regarding overlapping eligibility: approximately 17% to 42% of CLDN18.2-positive patients concurrently exhibit a high PD-L1 CPS ≥5, and roughly 14% to 15% are HER2-positive (37). The key question is what to do when both PD-L1 and CLDN18.2 are positive. At this time, there is insufficient data to indicate which treatment would be most effective for patients (38). Cohort 3B – zolbetuximab + pembrolizumab – was never included in the ILUSTRO study. Instead of continuing pembrolizumab, the study added cohort 4, which evaluated zolbetuximab + mFOLFOX + nivolumab as first-line treatment. Promising results led to the ongoing phase III LUCERNA study (39).

Notably, up to 52% of “triple-negative” tumors (HER2-negative, PD-L1-negative, and MMR-proficient) demonstrate CLDN18.2 positivity, increasing the overall proportion of patients with at least one targetable biomarker to an impressive 92% (37).

## 6 Limitations

Only two of the included studies were phase III RCTs; the rest were early-phase non-randomized or single-arm studies, which are inherently more susceptible to bias. The patient populations in the RCTs are also small, so the generalizability of the results may be limited. The FAST study used a different CLDN18.2 detection threshold than the SPOTLIGHT and GLOW trials, making inter – study comparisons difficult. Furthermore, the chemotherapy regimens varied across studies, making it difficult to isolate the effect of zolbetuximab alone on treatment and outcomes. Additionally, due to the significant clinical and methodological heterogeneity among the included trials—specifically regarding study designs, treatment lines, and biomarker thresholds—a meta-analysis could not be robustly performed, limiting the present study to a systematic evaluation of the outcomes. A larger proportion of study participants were from Asian countries compared to Western populations, which may limit generalizability. Responses to treatment are often variable. Importantly, clinical data on the quality of life of patients undergoing treatment were not provided, and in everyday clinical practice, patient experience is as important as treatment efficacy. Another important limitation is that both RCTs, which constitute the most important evidence of zolbetuximab effectiveness, were sponsored by Astellas Pharma, the manufacturer of Vyloy (zolbetuximab). Currently, there are no independent, investigator-initiated studies of zolbetuximab.

## 7 Conclusion

Zolbetuximab represents a notable advancement in the treatment of patients with locally advanced or metastatic G/GEJ adenocarcinoma, offering a highly specific targeted therapy directed against the CLDN18.2 protein. Pivotal phase III trials (SPOTLIGHT and GLOW) demonstrated that adding zolbetuximab to standard first-line chemotherapy significantly prolonged both PFS and OS in patients with high CLDN18.2 expression and HER2-negative status, expanding the therapeutic options for this group of patients.

The drug’s safety profile is acceptable, with the predominant adverse events of nausea and vomiting being primarily mild to moderate (grade 1–2) and most often resolving after initial treatment cycles. Phase I and II trials (Japanese Phase I, ILUSTRO, and FAST) provided evidence supporting safety, pharmacokinetics, and preliminary efficacy across various treatment settings and at varying CLDN18.2 expression thresholds. However, all included studies were industry-sponsored, and independent validation has not yet been conducted. Furthermore, the optimal positioning of zolbetuximab versus ICI-based regimens in patients with overlapping biomarker eligibility criteria (CLDN18.2 positivity and PD-L1 CPS ≥5) remains an important unresolved question.

Routine CLDN18.2 IHC testing using an FDA-approved companion diagnostic (VENTANA CLDN18 [43-14A] RxDx assay) should now be incorporated into the standard diagnostic workup for advanced G/GEJ adenocarcinoma.

## Data Availability

The original contributions presented in the study are included in the article/supplementary material. Further inquiries can be directed to the corresponding author.
